# Middlesex Hospital

**Published:** 1830-06

**Authors:** 


### HOSPITAL REPORTS.

### MIDDLESEX HOSPITAL.

### *Cases of Jaundice, with Disease of the Pancreas.*

John Pyemont, aged fifty, clerk to a surveyor, admitted Nov. 3, 1829, under Dr. Watson.

Skin and conjunctivæ of a deep yellow colour; bowels seldom moved; no stool for the last three days. Urine dark brown. Complains of debility and languor. States that he was taken ill, nearly six weeks ago, with pain in the head and loins; and was twice bled with great relief; that his bowels, previously regular, became sluggish at the commencement of his illness, and that the dejections from that period have resembled water gruel in colour and consistence; that he has been confined to bed for the last month, has been very feverish, and has wasted considerably; that he is now better in all respects, except the jaundice, which came on a week or ten days since. Objects do not appear yellow to him. Tongue rough, and covered with a dirty fur; says that within the last few days he has recovered the sense of taste, which he had lost during the former part of his illness. Pulse ninety-six, weak.

℞. Pilulæ Hydrargyri, Extracti Aloës purificati, āā gr. ijss.; Mucilag. q. s. M. fiat pilula, nocte maneque sumenda. Sumat etiam, ter quotidie, Magn. Sulphatis ʒi. ex Mist. Camph. f℥iss.

4th.—The abdomen was carefully examined, the patient being in bed. There is a visible fulness of the epigastrium on the right side, just beyond the cartilages of the upper false ribs: here a firm roundish tumor may be felt, about as large as a small apple, apparently circumscribed, and distant a quarter of an inch from the edge of the ribs. Further back, immediately beneath the ribs themselves, the abdomen is soft and supple. Says he suffers no pain in any part, nor does firm pressure on the projecting tumor occasion any uneasiness. Bowels not moved since his admission.

℞. Infusi Sennæ comp. f℥i.; Tr. Sennæ f℥ss.: Potassæ Tart. ʒij. M. fiat haustus, quam primum sumendus. Perstet in usu pilularum, et adhibeantur Hirudines decem epigastrio.

7th.—Thinks himself better since the application of the leeches. Bowels open; character of the stools unaltered. The urine, when collected in considerable quantity, appears almost black: dilution with water shows that this appearance is produced by concentration of the colouring matter of the bile; when much diluted, the urine becomes of a bright, transparent yellow.

Repetantur Hirudines vi., et sumat pilulam ter quotidie.

From this time to the 23d, on which day he died, he became progressively weaker, without any other remarkable change, except that, during the last few hours of his life, he was much harassed by nausea and retching, which had not occurred previously.

The body was examined on the 24th.

Universal yellowness of the surface, and great emaciation.

In the thorax, the right lung occupied a smaller space than ordinary, the diaphragm being pushed upwards by the liver, the upper surface of which was on a level with the upper edge of the fifth rib. The substance of the lungs was generally healthy; a hard portion at the summit of the right lung proved, when cut through, to be a moderately sized tubercle. No other tubercle was detected. The pleura covering that part of the right lung which lay undermost, and was fullest of blood, was marked by numerous small red spots, like those seen on the serous membranes in some cases of purpura.

The abdomen being opened, the tumor, which had been supposed to appertain to the liver, was found to be quite unconnected with it. It consisted of a mass of cancerous disease, disposed about the pyloric extremity of the stomach, involving in its central part the pancreas, and lying along the dorsal vertebræ. This mass was not homogeneous, nor very hard: it appeared to be composed of many distinct roundish tubera, pressed closely together, and of a dull yellowish colour. Several scattered tubera of the same kind were imbedded in the substance of the pancreas. The mucous membrane of the stomach and duodenum was entire, and apparently healthy; the pyloric orifice seemed slightly narrowed by the diseased mass which lay round it.

On the surface of the liver, and dispersed through its substance, there were numerous small, yellowish-white tubera, most of them about as big as a hazel nut; some having a curdy consistence, others softer and more fluid, resembling collections of very thick pus. They were not contained in cysts, but adhered loosely to the proper tissue of the liver. In the intervals between them, the liver itself was of a deep lead colour, and in other respects apparently healthy. The gallbladder was greatly distended; it contained a fluid which was tolerably limpid, and as black as ink. Dilution with a large quantity of water changed the black to a bright yellow colour. The cancerous mass which lay about the pylorus and duodenum had encircled, and completely closed, the extremity of the ductus communis choledochus.

There was a considerable hydatid in the pelvis of one kidney, and a smaller one in the cortical substance of the other. The colon was extremely narrow; and some of the folds of small intestines were adherent to each other.

The head was not examined.

Case II. Elizabeth Mounteney, aged fifty-four, admitted September 29th, 1829, under Dr. Watson.

Intense yellowness of the skin and conjunctivæ; urine very high coloured; dejections reported to be sometimes dark and sometimes clay coloured. The liver can be felt extending beyond the ribs, and partly across the epigastrium; its edge appears to be in some parts knotty and uneven: no pain, or a very slight sensation only of uneasiness, is produced by firm pressure upon this part. Abdomen elsewhere soft and natural. States that she was told, about a fortnight ago, that she was jaundiced, and was not previously aware of it; that she suffers no pain, and has suffered none; but has considered herself a healthy woman till about six months ago, when she began, without any known cause, to feel ill and uncomfortable. Since that time her appetite has failed, she has gradually wasted, and been languid and low spirited. She can lie without inconvenience in any position. Objects do not seem yellow to her. Bowels confined at present.

Sumat Pil. Hydrarg. cum Aloë gr. v. statim, et Haustum Sennæ comp. post horas quatuor.

30th.—Bowels freely purged; no trace of bile in the dejections. —Perstet in usu Pilulæ omni nocte. Capiat Magnesiæ Sulphatis ʒi. ex Inf. Rosæ comp. f℥ij. ter in dies.

October 5th.—No change. Bowels not purged; stools reported dark.—Omittantur medicamenta. ℞. Infusi Sennæ comp., Dec. Aloës comp. āā f℥ss. Fiat Haustus, ter quotidie sumendus.

On the 7th, she complained that the medicine occasioned griping pains in the bowels. It was omitted, and a decoction of Taraxacum substituted in its stead. After this, till near the end of the month, she remained free from pain, and even thought herself better: the jaundice, however, continued unaltered, the stools were pulpy and of a greyish blue colour, and she grew visibly thinner. In the early part of November, she began to suffer, at intervals, severe pain in the abdomen, which had become full and tympanitic: the pain occurred chiefly in the night, and opiates became necessary for its alleviation. The yellow colour of the skin assumed a slight tinge of green. She used at this time the nitro-muriatic bath for some days, without benefit. By degrees the abdominal pains became more frequent, and some tenderness was complained of when pressure was made upon the right hypochondrium; and she became more and more feeble and languid. In December, she began to experience pain at the epigastrium, of about half an hour's duration, immediately upon taking food, or drink, or medicine; and the pain was constant for a day or two before her death, which took place on the 14th.

The body was examined the next day.

The skin was universally yellow; the emaciation extreme.

The tympanitic condition of the abdomen had disappeared: its cavity being laid open, the liver was seen extending an inch or two below the ribs; just beyond its edge projected the distended gallbladder; and between this and the pyloric end of the stomach there was another prominence, in contact with the edge of the liver, and occasioned chiefly by a diseased condition of the pancreas. These were the irregularities which had been felt along the edge of the liver during the lifetime of the patient, and which had led to an erroneous conjecture that tubera existed in that viscus. The liver itself was perfectly sound, but of a deep yellow colour, both on its surface and internally. The gallbladder very large and tense; the cystic duct of its ordinary caliber; the hepatic and common duct so dilated, up to the junction of the latter with the duodenum, as to be capable of admitting the extremity of the little finger: they contained a black, tarlike, somewhat grumous fluid, which was not rendered yellow by dilution.

That part of the pancreas which extends across the vertebral column was sensibly indurated, and its central duct distended considerably beyond its natural size, the enlargement increasing towards the duodenum. The head of the pancreas, or that portion of it which is connected by cellular tissue to the duodenum, was in some parts as firm as cartilage, whilst the intervening parts were of the consistence of a scrofulous mesenteric gland: the harder portions were white, roundish, and of irregular surface; the softer yellow, and apparently rendered so by the colouring matter of the bile. The mucous membrane of the stomach and pylorus was sound; a little way within the duodenum, there was a circular depression, nearly as large as a shilling: here the several coats of the intestine were found to be entirely destroyed; and the handle of the scalpel passed, with very slight pressure, into the diseased head of the pancreas, which supplied the deficiency in the proper parietes of the intestine. The mucous membrane immediately surrounding the depression was sound; but the disease had extended to that portion of the gut where the biliary and pancreatic ducts pass obliquely between its tunics to terminate on its internal surface: here the intestine, throughout its entire substance, was thickened and scirrhous; and the excretory ducts of the liver and pancreas impervious.

The colon was small. Some portions of it were considerably smaller than others; one of these contracted portions was so short as to present the appearance of a distinct stricture. The coats of this intestine, in the more contracted portions, were thicker than elsewhere, and the mucous membrane much puckered.

The left ovary was as big as an orange; its surface was streaked by large veins, and in some parts of a deep purple colour. A section of it laid open numerous cysts, containing each a viscid, glairy fluid, which varied in colour, in different cysts, from a pale amber to a dark purple.

The thorax was sound. The aorta contained fluid and very thin blood.

John Hunter, after stating that bile, when mixed with the blood out of the body, prevents its coagulation, adds, "but we cannot suppose that in a living body it can be taken into the blood in such quantity as to produce the effect." Could it have been so in this case?

Disease of the pancreas, of which each of these cases furnishes an example, is spoken of as rare by all systematic writers on pathology. Andral's volume on the "Maladies of the Abdomen,"

contains one instance only of cancerous or other alteration of this gland. He remarks upon the infrequency of such morbid change, and says, with truth, that we generally find the pancreas untouched in the midst of the most extensive disorganization of the stomach, and of the tissues surrounding it. Dr. Baillie, in the volume of "Lectures and Observations on Medicine," printed after his death, states that the pancreas is, upon the whole, less liable to disease than any other important gland in the body. "I do not recollect," he says, "that in private practice I have met with one case in which there was satisfactory evidence of the pancreas being diseased; and I have only known of a solitary example of it during the thirteen years in which I was a physician of St. George's Hospital."

These cases afford also a sufficient illustration of the difficulty so often met with by the practical physician, of determining, from symptoms, the precise seat and nature of chronic disease within the abdomen; even when such disease has produced alterations of structure which are perceptible by the touch. The truth is that, of several of the abdominal viscera, the chronic diseases are marked by no *essential* symptoms, and that the *accidental* symptoms by which they may be accompanied relate as frequently to contiguous parts, as to the viscus primarily or principally affected.

---

